# Spatiotemporal epidemiology and SARIMA-based forecasting of hand, foot, and mouth disease outbreak clusters in Beijing, 2019–2024

**DOI:** 10.1186/s12879-026-13182-0

**Published:** 2026-03-26

**Authors:** Hao Zhao, Da Huo, Shuaibing Dong, Hui Xu, Zhiyong Gao, Jiaxin Feng, Renqing Li, Zhichao Liang, Yang Yang, Lei Jia, Xiaoli Wang, Peng Yang, Daitao Zhang

**Affiliations:** 1 Institute for Infectious Disease and Endemic Disease Control, Beijing Center for Disease Prevention and Control, No.16, Hepingli Middle Rd, Dongcheng District, Beijing, 100013 China; 2 Beijing Office of Global Health, Beijing Center for Disease Prevention and Control, Beijing, China; 3 School of Public Health, Capital Medical University, Beijing, China

**Keywords:** Hand, foot, and mouth disease, Outbreak clusters, Epidemiology, Pathogenesis

## Abstract

**Background:**

Hand, foot, and mouth disease (HFMD) is a common enteric infectious disease that poses a threat to children’s health. The disease exhibits high epidemic intensity and frequent outbreak clusters in Beijing, the capital of China. However, the spatiotemporal characteristics of these outbreak clusters and their future epidemic trends remain poorly understood. This study aims to fill this gap by analyzing the spatiotemporal epidemiology of HFMD outbreak clusters and developing a forecasting model to inform targeted prevention strategies.

**Methods:**

The present study analyzed data on HFMD outbreak clusters in Beijing from 2019 to 2024. Epidemiological characteristics were described, including demographic features, clinical manifestations, transmission settings, and spatial distribution. Group comparisons were performed using Kruskal-Wallis and chi-squared tests. The spatial distribution of outbreak clusters was visualized and analyzed by geographic region. A Seasonal Autoregressive Integrated Moving Average (SARIMA) model was developed to forecast incidence trends.

**Results:**

Beijing reported 4,265 HFMD outbreak clusters (12,396 cases) during 2019–2024, exhibiting a biennial high-incidence pattern. Compared to 2019 (1,206 clusters), the mean annual clusters during 2020–2022 decreased by 74.4% (305/year), rebounding to 1,719 clusters in 2023—42.5% higher than 2019. Most clusters (77.2%) involved < 5 cases. Median age was 5 years (IQR: 4–7), increasing to 6 years in 2023. Males predominated (*p* < 0.001). School-aged cases rose to 54.4% in 2023, while kindergarten cases declined. Kindergartens predominated overall (52.7%), but schools became the main setting in 2023 (44.9%), 4.2 times the pandemic average (10.6%). Clusters concentrated in near-suburban areas (> 50% in multiple years). CV-A6 was dominant (60.2%), peaking at 90.7% in 2023; EV-A71 was rare (0.12%). Cluster size varied by pathogen (*p* < 0.001). The SARIMA model forecast a bimodal pattern for 2025, with primary autumn peak (299 clusters) and secondary summer peak (165 clusters).

**Conclusion:**

HFMD outbreak clusters in Beijing exhibited a biennial pattern, with suppression during 2020–2022 due to COVID-19 NPIs followed by a rebound in 2023 exceeding pre-pandemic levels. Clusters concentrated in near-suburban areas with consistent bimodal seasonality, and the SARIMA model projects a similar pattern for 2025. These findings underscore the need for setting-specific strategies prioritizing schools alongside kindergartens, targeted interventions in high-risk suburbs, and continued pathogen monitoring.

## Introduction

Hand, foot, and mouth disease (HFMD) is a common infectious disease caused by infection with enteroviruses. It can be transmitted through direct or indirect routes, such as the fecal-oral route or via respiratory droplets. The disease primarily affects children < 5 years of age, who are more susceptible to severe complications due to their relatively lower immune levels [1]. Clinical manifestations primarily include fever and the appearance of rashes or blisters on the hands, feet, mouth, buttocks, and other areas. Most patients experience mild symptoms; however, a small number may develop aseptic meningitis, encephalitis, or myocarditis. In rare cases, severe illness in children can progress rapidly and may lead to death [2]. HFMD was first identified and named in New Zealand in 1957. Subsequently, large-scale outbreak clusters have occurred in various geographical regions, particularly in East and Southeast Asia [3–5]. In 2008, a severe outbreak of HFMD occurred in Anhui Province, China, primarily caused by EV-A71, resulting in tens of thousands of infections and > 100 deaths [6]. In May 2008, China classified HFMD as a Category C infectious disease for management [2].

Since 2008, HFMD cases have been reported annually in Beijing. In 2008, the number of cases exceeded 10,000, primarily affecting children < 5 years of age [7]. Initially, HFMD was primarily caused by EV-A71 and CV-A16 [8]. The Beijing municipal government and health authorities placed high importance on the disease, strengthening epidemic surveillance and prevention and control measures. Following the introduction of the EV-A71 vaccine in 2016, a marked decline in EV-A71-associated HFMD cases was observed. However, cases caused by CV-A6, CV-A16, and other enteroviruses continue to be prevalent [9, 10]. In recent years, the reported number of HFMD cases in Beijing still accounts for a relatively high proportion of notifiable infectious diseases [11]. A significant portion of this disease burden stems from frequent outbreak clusters, which predominantly affect young children. Schools and childcare centers remain high-risk settings for such outbreak clusters, posing ongoing threats to child health and regularly disrupting educational routines.

Despite their public health impact, systematic analyses of the epidemiological characteristics of HFMD outbreak clusters in Beijing—including seasonal patterns, cluster scale, regional distribution, etiological profiles, and population distribution—remain lacking. Furthermore, predictive models to assess the risk of these outbreak clusters have not yet been developed. Therefore, this study analyzed the epidemiological trends and etiological characteristics of HFMD outbreak clusters in Beijing from 2019 to 2024 and applied the Seasonal Autoregressive Integrated Moving Average (SARIMA) model to predict HFMD cluster incidence for 2025.

Compared with previous studies on HFMD in Beijing, this work presents several novel aspects. First, while earlier research primarily focused on overall HFMD incidence patterns [12, 13]. our study specifically examines outbreak clusters, which represent the most critical transmission units for targeted intervention. Second, Studies from multiple regions, including Mainland China [14] South Korea [15], and China’s Hong Kong [16], have indicated that public health and social measures (PHSMs)—such as wearing masks, frequent handwashing, regular ventilation, and maintaining safe social distancing—are likely associated with reduced transmissibility of hand, foot, and mouth disease (HFMD). we extend the temporal scope to 2019–2024, encompassing the COVID-19 pandemic period, thereby allowing an assessment of how unprecedented non-pharmaceutical interventions affected HFMD cluster dynamics. Third, we provide a detailed analysis of the etiological spectrum of clustered cases, revealing pathogen-specific patterns that may differ from general HFMD cases. Finally, to our knowledge, this is the first study to apply the SARIMA model specifically for forecasting HFMD cluster incidence in Beijing, offering a practical tool for public health preparedness and proactive response. The aim was to better understand the characteristics of HFMD outbreak clusters in Beijing and to implement preventive measures in advance to address potential epidemics in the coming year.

## Materials and methods

### Data source

Data on HFMD outbreak clusters and associated laboratory results were obtained from the Beijing HFMD Epidemiological Investigation Database, a centralized platform administered by the Beijing CDC. For each identified outbreak cluster, district-level CDCs are required to complete standardized epidemiological investigation forms and submit clinical specimens from affected cases for enterovirus testing. The investigation collects information including the time and settings of the outbreak, type of institution, demographic characteristics of cases, dates of illness onset, and medical attendance details.

For pathogen detection, at least two clinical specimens (throat swabs or stool samples) were collected from each outbreak cluster. These specimens were tested by district CDC laboratories using real-time reverse transcription polymerase chain reaction (RT‑PCR) for enterovirus serotyping, including EV‑A71, CV‑A16, CV‑A6, and other enteroviruses, following standardized protocols issued by the Beijing CDC.

All investigation records and corresponding laboratory results are entered into the epidemiological investigation database by district CDC staff. Each outbreak cluster is assigned a unique identifier upon initial entry, which links epidemiological information with laboratory test results within the same system. To ensure data quality and consistency, all submissions undergo review and validation by designated staff at the Beijing CDC before being finalized.

### Related definitions

#### Microbiological analysis

According to the Chinese Health Industry Standard for Diagnosis of Hand, Foot and Mouth Disease (WS 588–2018) [17], a laboratory-confirmed case was defined as a clinically diagnosed case meeting at least one of the following criteria:

1. Serological testing (IgM): Detection of EV-A71 or CV-A16 specific IgM antibodies in serum or cerebrospinal fluid using ELISA or equivalent methods.
2. Serological testing (IgG): A four-fold or greater rise in EV-A71 or CV-A16 specific IgG antibody titers between acute and convalescent serum samples, or seroconversion from negative to positive.
3. Nucleic acid detection: Detection of EV-A71 or CV-A16 specific nucleic acid from nasopharyngeal swabs, anal swabs, stool, vesicular fluid, cerebrospinal fluid, or autopsy specimens using RT-PCR or real-time RT-PCR.
4. Virus isolation: Isolation of EV-A71 or CV-A16 from clinical specimens using cell lines such as RD, HEp-2, or Vero, following standard virus culture protocols.

Virus isolation was performed using RD and HEp-2 cell lines according to the standard protocols described in Appendix C of WS 588–2018.

#### Definition of HFMD outbreak clusters

According to the Beijing HFMD and Herpangina Surveillance and Outbreak Response Work Plan (2024 Edition), an HFMD outbreak cluster is defined as follows: > 5 but < 10 cases occurring within 1 week in the same childcare facility, preschool, school, or other collective institution; ≥ 2 cases occurring in the same class (or dormitory); ≥ 3 but < 5 cases occurring in the same natural village/neighborhood committee; or ≥ 2 cases occurring within the same household.

These criteria are fully aligned with the national Protocol for Management of HFMD Clusters and Outbreaks (2012 Edition) [18] issued by the National Health Commission. Importantly, this definition remained consistent throughout the entire study period (2019–2024), as Beijing’s annual work plans—including the 2019 through 2024 editions—have all adhered to the same core criteria established in the 2012 national protocol. While the national protocol classifies events with ≥ 10 cases as an “outbreak,” this study employs a more sensitive cluster definition to capture the full spectrum of epidemic progression. Consequently, the term “outbreak cluster” as used herein encompasses the entire continuum from early clusters to full outbreaks.

#### Regional division of Beijing

Beijing comprises 16 administrative districts that can be categorized based on factors including geographical location, urban functional layout, economic development level, and population density in the central area (Districts of Dongcheng, Xicheng, Chaoyang, Haidian, Fengtai, and Shijingshan), nearby suburbs (Districts of Daxing, Tongzhou, Shunyi, Changping, Mentougou, Fangshan), and outer suburbs (Districts of Huairou, Pinggu, Yanqing and Miyun).

### Data processing and statistical analyses

Information regarding the outbreak clusters is summarized using spreadsheet software (Excel, Microsoft Corp., Redmond, WA, USA). Continuous variables, such as the age of cases within outbreak clusters, are presented as median with interquartile range (IQR), and heterogeneity across groups was assessed using the Kruskal–Wallis test. Categorical variables, including sex of cases and regional distribution of outbreak clusters, were compared using the chi‑squared test. Differences in cluster scale associated with different enterovirus types were also evaluated with the Kruskal–Wallis test, followed by post‑hoc pairwise comparisons where statistical significance was indicated. Variations in the distribution of cluster locations were examined using Fisher’s exact test. All statistical analyses were performed using SAS version 9.4 (SAS Institute, Cary, NC, USA). The regional distribution was visualized using ArcMap 10.6. The prediction of HFMD outbreak clusters in Beijing for 2025 was performed using R version 4.4.2 (R Core Team [2020]; R Foundation for Statistical Computing, Vienna, Austria).

### SARIMA model

SARIMA (Seasonal Autoregressive Integrated Moving Average) is a seasonal extension of the ARIMA model that captures periodic patterns in time series data. A SARIMA model is denoted as SARIMA(p, d, q)(P, D, Q)s, which consists of two components: a non-seasonal part with parameters p (autoregressive order), d (differencing order), and q (moving average order); and a seasonal part with parameters P (seasonal autoregressive order), D (seasonal differencing order), and Q (seasonal moving average order), where s represents the length of the seasonal period (e.g., s = 12 for monthly data with an annual cycle) [19].

The time series modeling procedure comprised the following steps: First, stationarity of the monthly time series (January 2019 to December 2024) was assessed using the Augmented Dickey-Fuller (ADF) test. As the original series was non-stationary (*p* > 0.05), seasonal differencing was applied. Second, candidate model parameters were preliminarily identified by examining the autocorrelation function (ACF) and partial autocorrelation function (PACF) plots of the stationary series. Multiple SARIMA models with different parameter combinations were fitted and compared. Third, the optimal model was selected based on the lowest Akaike Information Criterion (AIC) and Bayesian Information Criterion (BIC). Model adequacy was verified using the Ljung-Box test on residuals to ensure no significant autocorrelation remained (*p* > 0.05). Finally, the selected model was used to generate out-of-sample forecasts for the 12 months of 2025, with 95% confidence intervals calculated to quantify prediction uncertainty.

### Model comparison

To ensure the robustness of our forecasting approach, we compared SARIMA with an alternative method. Both SARIMA and Holt-Winters exponential smoothing models were fitted to the HFMD outbreak cluster data. However, due to the presence of zero-case months in the original monthly series (which violates the non-negative requirement of Holt-Winters), the data were aggregated to quarterly intervals for Holt-Winters modeling. While the quarterly Holt-Winters model yielded a lower AIC (268.54) compared to the monthly SARIMA model (724.54), these values are not directly comparable due to differences in data frequency and sample size. The SARIMA model was ultimately selected for final forecasting because: (1) it preserves the original monthly resolution, which is more informative for outbreak detection and public health response; (2) it can appropriately handle zero values in the time series; and (3) its residuals satisfied white noise tests (Ljung-Box *p* = 0.682), indicating adequate model fit.

## Results

**Fig. 1:**
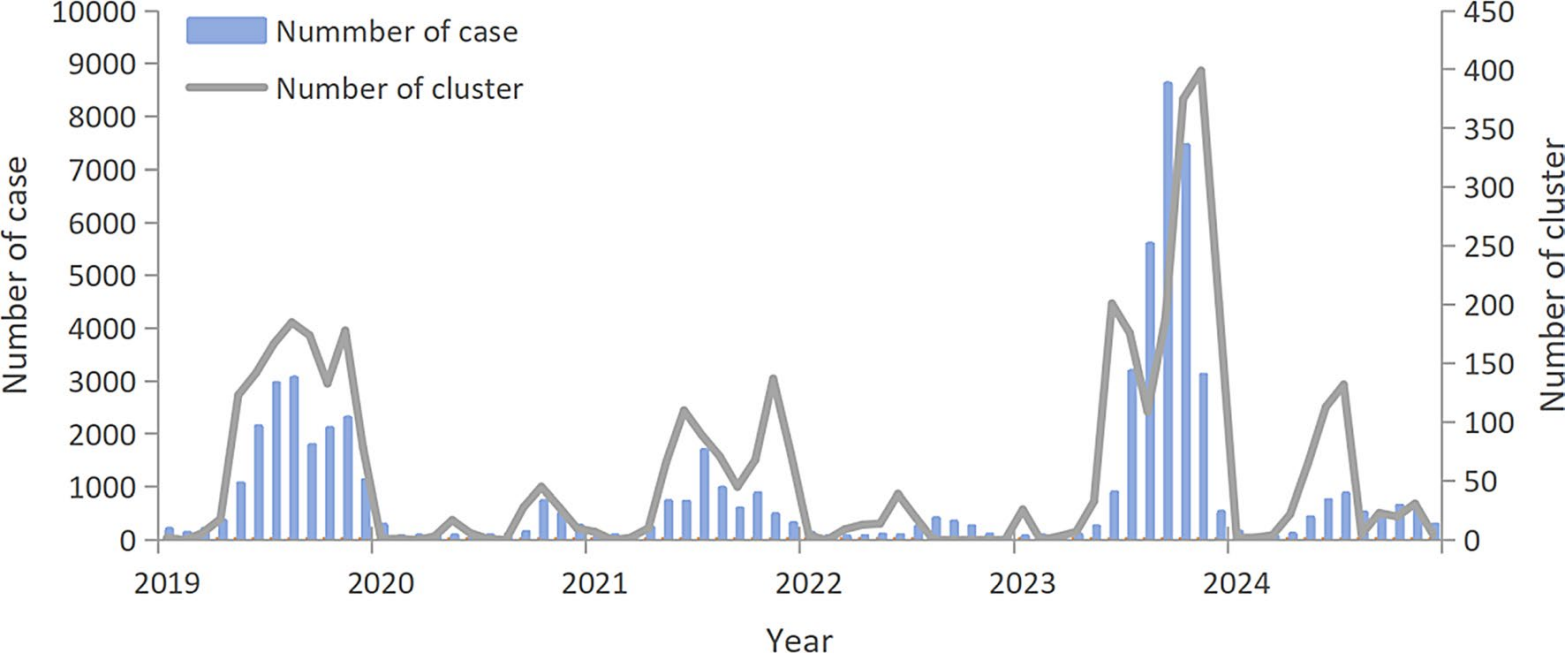
Monthly distribution of HFMD cases and outbreak clusters in Beijing, 2019-2024

From 2019 to 2024, Beijing reported 4,265 HFMD outbreak clusters, with an average of 710 outbreak clusters annually. Compared with the pre-pandemic level in 2019 (1,206 clusters), the mean annual number of outbreak clusters during 2020–2022 decreased by 74.4% (mean: 305 clusters/year), before rebounding to 1,719 outbreak clusters in 2023 − 42.5% higher than the 2019 level. Of the 4265 outbreak clusters, those involving < 5 cases, 5–9 cases, 10–19 cases, and ≥ 20 cases accounted for 77.2% (3291/4265), 11.2% (476/4265), 10.5% (446/4265), and 1.2% (52/4265), respectively. The trends in the number of reported HFMD cases and outbreak clusters in Beijing from 2019 to 2024 were consistent, exhibiting an alternating high-incidence pattern every other year (Fig. 1). The median interval between the onset date of the first case and the reporting date was 4 days, and the median interval between the onset dates of the first and last cases was 3 days.

From 2019 to 2024, the highest number of outbreak clusters occurred in 2023, whereas the lowest occurred in 2022. The monthly number of outbreak clusters peaked at 714 in September 2023 and dropped to 0 in some months. Seasonally, the cluster peaks mainly occurred in summer and autumn. Bimodal patterns were observed, except in 2020 and 2022, which did not show significant peaks. The annual peak months were November 2019, November 2020, July 2021, September 2022, and June 2023 (Fig. 1).

To identify the optimal forecasting approach, both SARIMA and Holt-Winters exponential smoothing models were fitted to the monthly HFMD outbreak cluster data from 2019 to 2024. Among the candidate SARIMA models, SARIMA(2,0,0)(0,1,1)₁₂ yielded the lowest Akaike Information Criterion (AIC = 724.54) and was therefore selected as the optimal model for forecasting (Table 1). The Ljung-Box test confirmed that the residuals of the selected model were white noise (*p* = 0.682), indicating adequate model fit. The model demonstrated acceptable predictive performance, with a root mean square error (RMSE) of 72.06 and a mean absolute error (MAE) of 29.71.This multi-indicator approach, encompassing information-based criteria (AIC), residual diagnostics (Ljung-Box), and error metrics (RMSE, MAE), aligns with recent recommendations for comprehensive evaluation of infectious disease forecasting models [20]. The MAE indicates that the typical forecast error was approximately 30 outbreaks per month, representing about 4% of the projected annual total. The mean absolute percentage error (MAPE) was 208.11%; this high value is attributable to the inclusion of the COVID-19 pandemic years (2020–2022) in the study period, during which intensive non-pharmaceutical interventions led to several months with extremely low outbreak counts—a known limitation of MAPE for time series containing near-zero values.

**Table 1:** AIC and BIC values for candidate SARIMA models

Candidate Models	AIC	BIC	Ljung-Box *p*-value
SARIMA(2,0,0)(0,1,1)₁₂	724.54	732.92	0.682
SARIMA(0,0,1)(0,1,1)	729.06	735.35	0.036
SARIMA(1,0,0)(1,1,0)₁₂ with drift	726.38	732.66	0.246
SARIMA(1,0,1)(0,1,1)₁₂	724.82	733.2	0.643
SARIMA(2,0,1)(0,1,1)	726.52	736.99	0.691

**Fig. 2:**
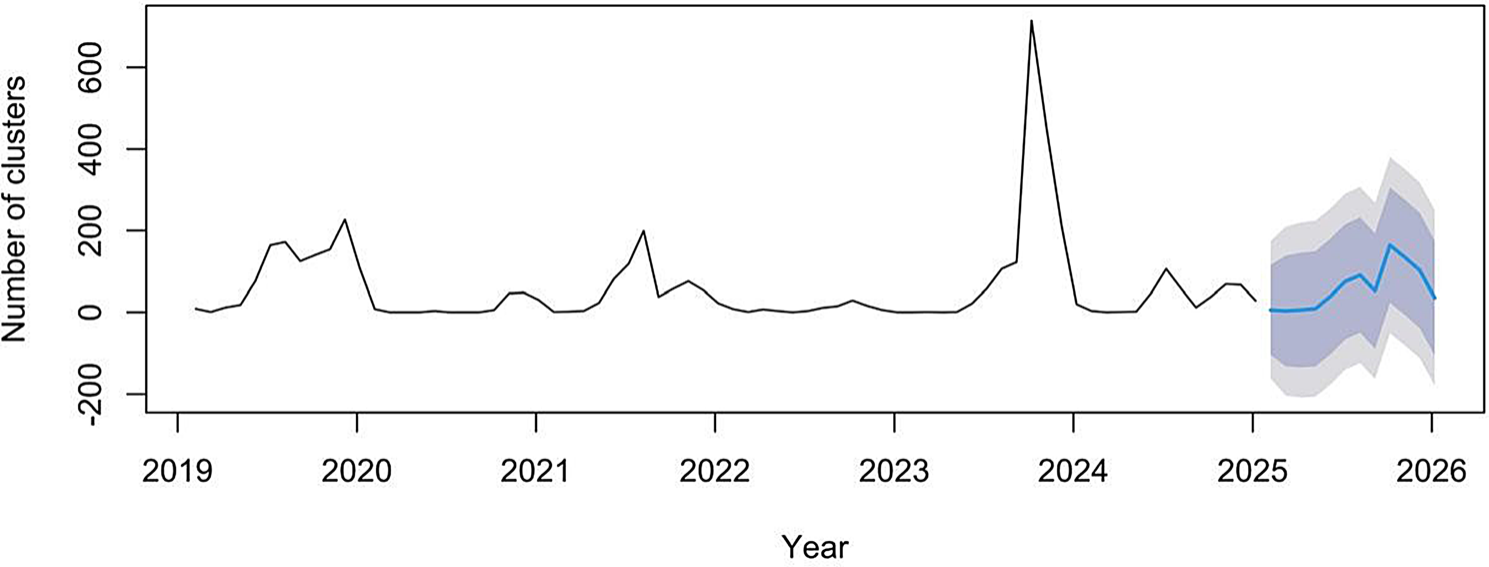
Monthly distribution of HFMD outbreak clusters during 2019-2024 and the forecast in 2025. Note: The blue line represents the forecast of the monthly distribution of HFMD outbreak clusters in 2025. The light gray shaded area denotes the 80% confidence interval, and the dark gray area represents the 95% confidence interval

Using this model, the total number of HFMD outbreak clusters in Beijing for 2025 was forecasted to be 716. A bimodal seasonal pattern was projected, with a primary peak in autumn (September–October), estimated at 299 outbreak clusters, and a secondary peak in early summer (June–July), estimated at 165 outbreaks (Fig. 2). These forecasts suggest that the epidemiological pattern observed in previous years will persist into 2025.

Among the 12,396 cluster-associated cases, the median age was 5 years (IQR: 4–7 years). Median age increased notably in 2023 (6 years) and 2024 (5 years), compared to a stable median of 4 years during 2019–2022. Male cases predominated in most years, although the sex distribution also differed significantly (*p* < 0.001). The population composition of cases shifted over time. The proportion of kindergarten children remained high until 2021 but decreased substantially in 2023 and 2024, whereas the proportion of school-aged students rose markedly in these two years, reaching 54.4% in 2023. Mild clinical manifestations were common throughout the period, with fever and vesicles being the most frequently reported symptoms. The prevalence of these symptoms also exhibited significant variation across years (*p* < 0.001)(Table 2).

**Table 2:** Demographic characteristics of HFMD outbreak cluster cases in Beijing during 2019 to 2024

Characteristics	2019 (*N* = 3522)	2020 (*N* = 357)	2021 (*N* = 2095)	2022 (*N* = 236)	2023 (*N* = 4762)	2024 (*N* = 1424)	*p*
**Age**,** year**,** median(IQR)**	4(3–5)	4(3–5)	4(4–5)	4(3–6)	6(4–9)	5(4–7)	< 0.001^a^
**Sex**,** n(%)**							< 0.001^b^
Boy	2065(58.6)	190(53.2)	1191(56.8)	139(58.9)	2952(62.0)	776(54.5)	
Girl	1457(46.8)	167(46.8)	904(43.2)	97(41.1)	1810(38.0)	648(45.5)	
**Population type**,** n(%)**							< 0.001^b^
scattered children	307(8.7)	26(7.3)	125(6.0)	33(14.0)	260(5.5)	15(1.1)	
Kindergarten children	2469(70.1)	256(71.7)	1598(76.3)	153(64.8)	1775(37.3)	926(65.0)	
Student	649(18.4)	62(17.4)	349(16.7)	46(19.5)	2591(54.4)	466(32.7)	
Others	97(2.8)	13(3.6)	23(1.1)	4(1.7)	136(2.9)	17(1.2)	
**Symptoms**							
Mild symptoms	3088(87.7)	344(96.4)	1909(91.1)	230(97.5)	4332(91.0)	1363(95.7)	
Pyrexia	2086(59.2)	232(65.0)	1338(63.9)	145(61.4)	3858(81.0)	942(66.2)	
vesicles	2991(84.9)	327(91.6)	1834(87.5)	221(93.6)	4013(84.3)	1110(77.9)	

IQR: Interquartile range

^a^Kruskal-Wallis test

^b^Chi-square test

Note: The comparison of the demographic characteristics of HFMD outbreak cases across different years

Kindergartens accounted for the highest proportion overall (52.7%), followed by schools (29.4%). However, a striking shift occurred in 2023, when schools became the predominant location, constituting 44.9% (772/1719) of all outbreak clusters for that year. This proportion was 4.2 times higher than the pandemic period average of 10.6% during 2020–2022. Concurrently, the proportion in kindergartens dropped to 31.8% in 2023, below the pandemic period average of 53.4%. The settings of HFMD outbreak clusters changed markedly over the study period (*p* < 0.001) (Table 3). Kindergartens were the most common location in most years, accounting for over 55% of outbreak clusters from 2019 to 2021 and in 2024. However, a striking shift occurred in 2023, when schools became the predominant location, constituting 44.9% (772/1719) of all outbreak clusters for that year, compared to a range of 9.2% to 13.1% in the preceding years. Concurrently, the proportion of outbreak clusters in households and communities remained relatively low across all years. The geographic distribution of outbreak clusters across Beijing’s districts also showed significant heterogeneity (*p* < 0.001)(Table 3). The central areas and near suburbs together accounted for the majority of outbreak clusters (approximately 90% or more) in each year. A notable pattern was observed in the near suburbs, which represented over 50% of outbreak clusters in 2020, 2021, 2022, and 2023, peaking at 67.3% (66/98) in 2022. The proportion of outbreak clusters located in other suburbs was comparatively lower throughout the surveillance period.

**Table 3:** Characteristics of HFMD outbreak cluster in Beijing during 2019 to 2024

Variables	2019(*N* = 1206)	2020(*N* = 140)	2021(*N* = 678)	2022(*N* = 98)	2023(*N* = 1719)	2024(*N* = 424)	*p*
**Setting**,** n(%)**							< 0.001^a^
Kindergarten	695(57.6)	78(55.7)	417(61.5)	42(42.9)	547(31.8)	262(61.8)	
School	158(13.1)	16(11.4)	76(11.2)	9(9.2)	772(44.9)	107(25.2)	
Household	349(28.9)	44(31.4)	173(25.5)	45(45.9)	383(22.3)	52(12.3)	
Community/Others	4(0.3)	2(1.4)	12(1.8)	2(2.0)	17(1.0)	3(0.7)	
**Region**,** n(%)**							< 0.001^b^
Central area	540(44.8)	60(42.9)	254(37.5)	27(27.6)	561(32.6)	183(43.2)	
Near suburbs	542(44.9)	75(53.6)	357(52.7)	66(67.3)	910(52.9)	203(47.9)	
Other suburbs	124(10.3)	5(3.6)	67(9.9)	5(5.1)	248(14.4)	38(9.0)	

^a^Fisher test

^b^Chi-square test

Note: The comparison of the distribution characteristics of HFMD outbreak cluster settings and geographic patterns across different years

**Fig. 3:**
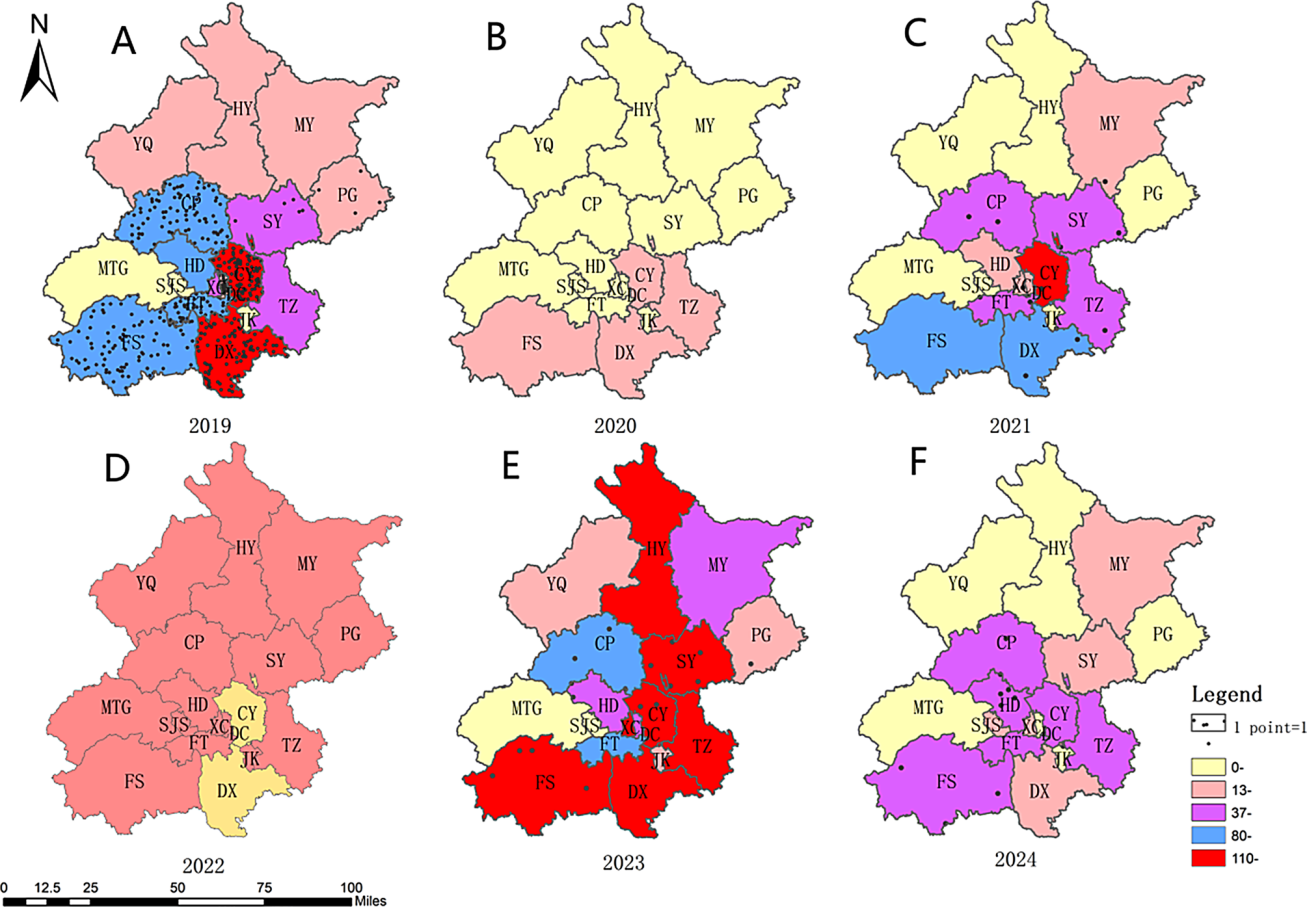
Spatial distribution of HFMD outbreak clusters in Beijing during 2019-2024. (**A**) Spatial distribution of HFMD outbreak clusters in 2019. (**B**) Spatial distribution of HFMD outbreak clusters in 2020. (**C**) Spatial distribution of HFMD outbreak clusters in 2021. (**D**) Spatial distribution of HFMD outbreak clusters in 2022. (**E**) Spatial distribution of HFMD outbreak clusters in 2023. (**F**) Spatial distribution of HFMD outbreak clusters in 2024. Abbreviations: DC, Dongcheng; XC, Xicheng; CY, Chaoyang; HD, Haidian; FT, Fengtai; SJS, Shijingshan; DX, Daxing; TZ, Tongzhou; SY, Shunyi; CP, Changping; MTG, Mentougou; FS, Fangshan; HR, Huairou; PG, Pinggu; MY, Miyun; YQ, Yanqing). Note: Dots represent the outbreak clusters involving more than 10 cases. Central area (Districts of DC, XC, CY, HD, FT, SJS), Nearby suburbs (Districts of DX, TZ, SY, CP, MTG, FS), Outer suburbs (Districts of HR, PG, YQ MY)

From 2019 to 2024, the districts with the highest number of outbreak clusters were Chaoyang (707), Daxing (614), and Fangshan (480). The district with the fewest outbreak clusters was Mentougou (10 outbreak clusters). In 2020 and 2022, the number of outbreak clusters was relatively low due to the impact of the coronavirus disease 2019 (COVID-19) pandemic, with no significant differences among the districts (Fig. 3(B)(D)). In 2019, 2021, 2023 and 2024, except for the Mentougou District, the number of outbreak clusters in the central area and near the suburbs was significantly higher than that in the outer suburbs (Fig. 3(A)(C)(E)(F)). The number of outbreak clusters in the Fangshan and Huairou districts exceeded 100 by 2023.

**Fig. 4:**
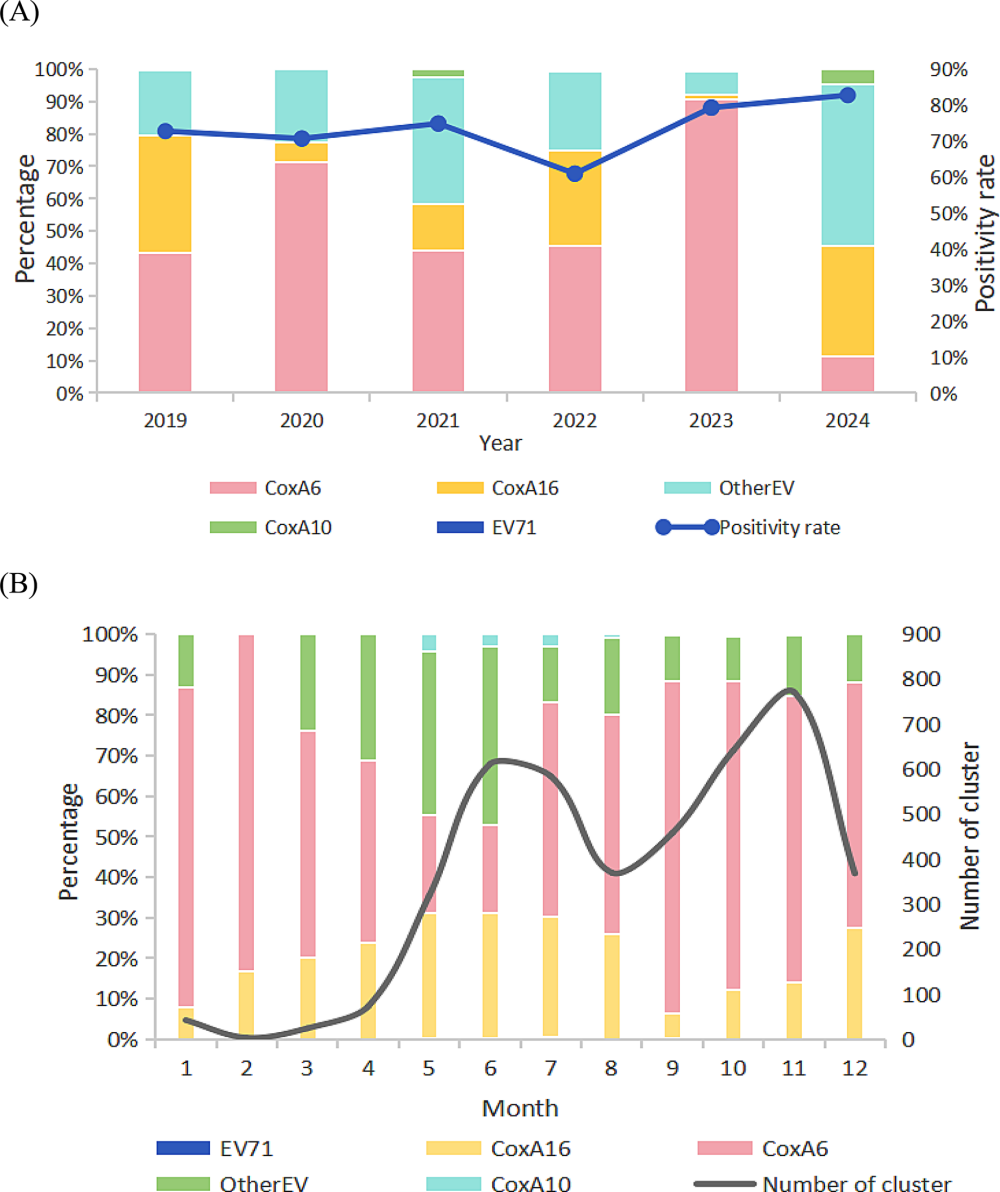
Pathogen’s spectrum of HFMD outbreak clusters in Beijing during 2019–2024. (**A**) The Pathogen spectrum and positivity rate of HFMD outbreak clusters. (**B**) The monthly distribution of HFMD outbreak clusters and pathogen spectrum

A total of 9767 specimens from cluster-related specimens were collected, corresponding to a positivity rate of 76.4% (7466/9767). Among the positive samples, the proportion of each subtype was as follows: CV-A6 (60.2%), other enteroviruses (21.7%), CV-A16 (16.9%), CV-A10 (1.2%), and EV-A71 (0.12%). The year with the highest positivity rate was 2024 (82.8% [840/1015]), while the year with the lowest positivity rate was 2022 (61.0% [128/210]) (Fig. 4A). Except for 2024, when other enteroviruses accounted for 50%, the dominant strain was CV-A6, with its proportion reaching its highest value (90.7%) in 2023. Both CV-A16 and CV-A6 were present in all years. Monthly data revealed that the proportion of CV-A6 initially decreased and then increased, reaching its lowest point in June (21.6%), and gradually rising to its peak in November (70.8%) (Fig. 4B). In contrast, the proportions of CV-A16 and other enteroviruses exhibited the opposite trend, initially increasing and then decreasing.

The Kruskal–Wallis test was used to statistically compare differences in the number of cases caused by EV-A71, CV-A16, CV-A6, and other enteroviruses. The results revealed that the overal differences in cluster scale caused by the four types were statistically significant (χ²= 137.32, *P* < 0.001)(Table 4). Further pairwise comparisons revealed that these differences were mainly reflected in the cluster scales caused by CV-A16, CV-A6 (*P* < 0.001), CV-A6, and other enteroviruses (*P* < 0.001).

**Table 4:** Outbreak cluster scale caused by pathogen spectrum

Pathogen spectrum	EV71	CoxA16	CoxA6	coxA10	OtherEV	*P*
**Number of cases (median**,** IQR)**	4(2,12)	3(2,9)	2(2,3)	2(2,4)	3(2,5)	< 0.001^a^

IQR: Interquartile range

^a^Kruskal-Wallis test

## Discussion

This study integrates spatiotemporal epidemiological analysis with SARIMA-based forecasting to provide a comprehensive assessment of HFMD outbreak clusters in Beijing from 2019 to 2024, revealing several key findings with important public health implications. The long-term epidemic dynamics of HFMD outbreak clusters in Beijing from 2019 to 2024, which revealed a biennial high-incidence pattern, consistent with the overall trend of HFMD in the city. Research suggests that the biennial cycle of HFMD epidemics may be related to changes in the dominant circulating strains [21]. Notably, incidence remained relatively low between 2020 and 2022, likely due to the comprehensive non-pharmaceutical interventions (NPIs) implemented for COVID-19 prevention and control, including mask-wearing, frequent handwashing, ventilation, and social distancing [22].This disruption was not unique to Beijing. Studies from Shanghai and Zhejiang [23, 24]. Province also reported substantial declines in HFMD cases during 2020–2021 compared to pre-pandemic levels, as well as altered seasonal epidemic peaks. These consistent findings across different geographic settings demonstrate the profound impact of broad-spectrum NPIs on enterovirus transmission.

The subsequent increase in HFMD outbreak clusters in Beijing during 2023 was characterized by a prominent autumn peak, with the annual number surging to 1,719 clusters—42.5% higher than the 2019 pre-pandemic level. This resurgence likely reflects two concurrent factors: the resumption of normal social interactions facilitating transmission among children [25], and a potentially expanded susceptible pool due to reduced viral exposure during the pandemic. However, given that enterovirus immunity is serotype-specific and transient [26], the concept of “immunity debt” is less straightforward for HFMD than for respiratory infections. The delayed but intensified epidemic rebound observed in Beijing highlightsz the complex interplay between non-pharmacological interventions, population immunity, and enterovirus transmission dynamics.

HFMD outbreak clusters in Beijing from 2019 to 2024 displayed consistent bimodal seasonality (summer and autumn peaks), the pronounced seasonal characteristics suggest a potential influence from climatic factors, such as temperature and humidity. This hypothesis is supported by a recent study in Fuzhou, China [27]. This bimodal pattern aligns with findings from France, where HFMD outbreaks also showed summer-autumn bimodality [28]. In contrast, England exhibits a single annual peak [29], suggesting geographic variation in seasonal drivers possibly related to climatic or population factors.

The SARIMA model further predicted a distinct biphasic pattern for 2025: a smaller, delayed spring peak followed by a larger autumn peak at the typical timing. If this pattern actually plays out, it might offer some practical takeaways for public health planning. For example, the delayed spring peak could mean that routine prevention measures, like school hygiene campaigns or environmental cleaning, might not need to ramp up quite as early as in typical years. On the other hand, the projected larger autumn peak suggests it would be wise to ensure that surveillance and response capacity are ready to go by August, just in case. In short, rather than providing definitive answers, this prediction offers a rough roadmap for preparedness: public education efforts could be tentatively planned in two waves aligned with the potential peaks, with messaging tailored to school settings before the autumn semester—while remaining flexible enough to adjust as real-time data come in.

The median age of cases involved in outbreak clusters was 5 years, significantly higher than that of overall HFMD cases in Beijing [30]. The median age showed an annual increasing trend, reaching 6.7 years in 2023. This rise is likely multifactorial. While the lifting of COVID-19 restrictions and resumption of regular schooling in 2023 may have played a role [31, 32], other factors may have contributed as well. Data from the Beijing Statistical Yearbook show that the age composition of Beijing’s child population (0–14 years) remained relatively stable between 2022 and 2023 [33], suggesting that demographic shifts are unlikely to be the primary driver. However, changes in kindergarten enrollment patterns post-pandemic, or age-specific differences in healthcare-seeking behavior and reporting practices, cannot be ruled out and warrant further investigation. The male-to-female ratio peaked in 2023, despite stable sex ratios in Beijing’s child population [33]. This pattern aligns with England’s [29].general practitioner consultation data showing higher rates among males, and may reflect behavioral differences: male children typically engage in more physical activity and higher-risk interactions, potentially increasing their susceptibility following school reopenings, a finding consistent with previous studies [34, 35].

In terms of transmission settings, kindergartens were higher-risk settings for outbreak clusters compared with schools, which may be related to the higher susceptibility among younger children [36]. The majority (77.2%) of outbreak clusters involved < 5 cases, indicating that most outbreak clusters likely occurred primarily within individual classes. Therefore, it is crucial to secure a “classroom” as a key control point—containing the transmission chain within the classroom helps curb further spread. Given that asymptomatic carriers are a major risk factor for HFMD transmission in schools and kindergartens [37], enhancing ventilation, disinfection, frequent hand washing, and maintaining hygiene within classrooms are crucial measures for preventing cluster spread. Studies have shown that implementing zoned and grade-segregated teaching in childcare institutions can help control HFMD outbreak clusters [38]; it is recommended that kindergartens establish reasonable teaching mechanisms and improve educational environments. Regarding regional distribution, the characteristics of HFMD outbreak clusters in Beijing are consistent with previous research findings [30]. The pattern shows no significant annual variation: near suburbs> central areas> outer suburbs, likely reflecting population density and migration patterns. This suggests that prevention efforts should prioritize urban–rural junctions and areas with dense migrant populations.

CV-A6 was the dominant pathogen throughout the study period, peaking at 90.7% in 2023, while EV-A71 was rare. This dominance aligns with evidence that CV-A6 has pandemic potential in Europe [39]. Compared with HFMD caused by CV-A16 and other enteroviruses, the outbreak clusters caused by CV-A6 were relatively smaller, possibly reflecting its high transmissibility but lower virulence [24].The opposite seasonal trends between CV-A6 and CV-A16/other enteroviruses suggest serotype-specific differences in environmental or host factors. Methodological differences in viral typing across Chinese regions (e.g., CV-A6 classified as “other enteroviruses” in Guangdong [40] and Qingdao [41]) highlight the need for standardized surveillance to enable meaningful inter-regional comparisons.

This study has several limitations. First, the COVID-19 pandemic (2020–2022) led to intensive non-pharmaceutical interventions that substantially altered HFMD transmission; thus, epidemiological patterns during this period may not reflect typical dynamics. Second, mandatory public health interventions (e.g., class suspension) upon cluster identification may have interrupted transmission, potentially leading to underestimation of true cluster size and progression. Third, while SARIMA is suitable for linear forecasting, it may not capture complex nonlinear relationships; future studies could explore machine learning approaches (e.g., LSTM). Fourth, missing denominator data for some schools and kindergartens may introduce selection bias, although no significant differences were observed between included and excluded cases.

## Conclusion

This study integrates spatiotemporal epidemiological analysis with SARIMA-based forecasting to characterize HFMD outbreak clusters in Beijing from 2019 to 2024. Several key findings emerged. First, outbreak clusters exhibited a biennial high-incidence pattern, with transmission substantially suppressed during 2020–2022 due to COVID-19 NPIs (74.4% decline vs. 2019), followed by a marked resurgence in 2023 that exceeded pre-pandemic levels by 42.5%. Second, clusters displayed consistent bimodal seasonality (summer and autumn peaks), aligning with findings from France but contrasting with England’s single annual peak, suggesting geographic variation in seasonal drivers. The SARIMA model projects a similar bimodal pattern for 2025, providing a roadmap for preparedness. Third, a notable epidemiological shift occurred in 2023–2024, characterized by increased median age and a transition from kindergartens to schools as the predominant transmission setting, with school-based clusters increasing 4.2-fold versus the pandemic period. Fourth, CV-A6 remained the dominant pathogen throughout, peaking at 90.7% in 2023, while EV-A71 was rare. Finally, clusters concentrated in near-suburban areas and urban–rural junctions, informing targeted prevention strategies. These findings underscore the need for setting-specific interventions, enhanced surveillance in high-risk areas, and continued monitoring of pathogen evolution.

## Data Availability

The analytical data in this study were sourced from the Beijing Center for Disease Control and Prevention(Beijing CDC) surveillance system and cannot be publicly disclosed due to privacy protection regulations. De-identified data are available from the corresponding author upon request.

## Abbreviations

HFMD: Hand, foot, and mouth disease
SARIMA: Seasonal Autoregressive Integrated Moving Average
PHSMs: Public health and social measures
CDC: Centers for Disease Control and Prevention
IQR: Interquartile range
NPIs: Non-pharmaceutical interventions
